# Study on a Novel Cold-Active and Halotolerant Monoacylglycerol Lipase Widespread in Marine Bacteria Reveals a New Group of Bacterial Monoacylglycerol Lipases Containing Unusual C(A/S)HSMG Catalytic Motifs

**DOI:** 10.3389/fmicb.2020.00009

**Published:** 2020-01-23

**Authors:** Ping-Yi Li, Yan-Qi Zhang, Yi Zhang, Wen-Xin Jiang, Yan-Jun Wang, Yi-Shuo Zhang, Zhong-Zhi Sun, Chun-Yang Li, Yu-Zhong Zhang, Mei Shi, Xiao-Yan Song, Long-Sheng Zhao, Xiu-Lan Chen

**Affiliations:** ^1^State Key Laboratory of Microbial Technology, Marine Biotechnology Research Center, Shandong University, Qingdao, China; ^2^Department of Hematology, Qilu Hospital, Shandong University, Jinan, China; ^3^College of Marine Life Sciences, Institute for Advanced Ocean Study, Ocean University of China, Qingdao, China; ^4^Laboratory for Marine Biology and Biotechnology, Pilot National Laboratory for Marine Science and Technology (Qingdao), Qingdao, China

**Keywords:** α/β hydrolase, monoacylglycerol lipase, marine bacterium, cold-adapted enzyme, halotolerance

## Abstract

Monoacylglycerol lipases (MGLs) are present in all domains of life. However, reports on bacterial MGLs are still limited. Until now, reported bacterial MGLs are all thermophilic/mesophilic enzymes from warm terrestrial environments or deep-sea hydrothermal vent, and none of them originates from marine environments vastly subject to low temperature, high salts, and oligotrophy. Here, we characterized a novel MGL, GnMgl, from the marine cold-adapted and halophilic bacterium *Glaciecola nitratireducens* FR1064^T^. GnMgl shares quite low sequence similarities with characterized MGLs (lower than 31%). GnMgl and most of its bacterial homologs harbor a catalytic Ser residue located in the conserved C(A/S)HSMG motif rather than in the typical GxSxG motif reported on other MGLs, suggesting that GnMgl-like enzymes might be different from reported MGLs in catalysis. Phylogenetic analysis suggested that GnMgl and its bacterial homologs are clustered as a separate group in the monoglyceridelipase_lysophospholipase family of the Hydrolase_4 superfamily. Recombinant GnMgl has no lysophospholipase activity but could hydrolyze saturated (C12:0-C16:0) and unsaturated (C18:1 and C18:2) MGs and short-chain triacylglycerols, displaying distinct substrate selectivity from those of reported bacterial MGLs. The substrate preference of GnMgl, predicted to be a membrane protein, correlates to the most abundant fatty acids within the strain FR1064^T^, suggesting the role of GnMgl in the lipid catabolism in this marine bacterium. In addition, different from known bacterial MGLs that are all thermostable enzymes, GnMgl is a cold-adapted enzyme, with the maximum activity at 30°C and retaining 30% activity at 0°C. GnMgl is also a halotolerant enzyme with full activity in 3.5M NaCl. The cold-adapted and salt-tolerant characteristics of GnMgl may help its source strain FR1064^T^ adapt to the cold and saline marine environment. Moreover, homologs to GnMgl are found to be abundant in various marine bacteria, implying their important physiological role in these marine bacteria. Our results on GnMgl shed light on marine MGLs.

## Introduction

Lipases (EC 3.1.1.3) are a group of α/β hydrolases that catalyze the hydrolysis of ester bonds in water-insoluble long-chain acylglycerols. Monoacylglycerol lipases (MGLs) (EC 3.1.1.23) are a subclass of lipases, which specifically catalyze the hydrolysis of monoacylglycerol with low stereo-selectivity. MGLs are widely distributed in bacteria, yeast, plant, and mammals. In the ESTHER (ESTerases and alpha/beta-Hydrolase Enzymes and Relatives) database (Lenfant et al., 2013), MGLs are classified into (super)families Pancreatic_lipase, α,β-hydrolase domain-containing protein 6 (ABHD6), ABHD16, Duf_676, and Hydrolase_4 based on amino acid sequence similarity.

Most reported MGLs are from mammals and their physiological roles in mammals are well-studied. Mammalian MGLs play an essential role in energy homeostasis by catalyzing the last step of intracellular phospholipid and triacylglycerol degradation (Fredrikson et al., 1986; Taschler et al., 2011). MGLs also participate in mediating endocannabinoid-based signaling in mammalian brains to regulate processes such as pain, inflammation, memory, and cancer (Hohmann et al., 2005; Nomura et al., 2010; Micale et al., 2013), thus making them important pharmacological targets (Gil-Ordóñez et al., 2018).

In contrast to the extensive study of MGLs in mammals, only several bacterial MGLs have been biologically characterized in detail, including a MGL from soil bacterium *Pseudomonas* sp. LP7315 (Sakiyama et al., 2001) (named as pMGL in this study), bMGL from moderately thermophilic soil bacterium *Bacillus* sp. H257 (Imamura and Kitaura, 2000; Kitaura et al., 2001), LipS from a soil metagenomic library (Chow et al., 2012; Riegler-Berket et al., 2018), GMGL from *Geobacillus* sp. 12AMOR1 isolated from a deep-sea hydrothermal vent (Tang et al., 2019), MSMEG_0220 from human non-pathogenic *Mycobacterium smegmatis* (Dhouib et al., 2010) and Rv0183 from human pathogenic *Mycobacterium tuberculosis* H37Rv (Cotes et al., 2007), which all belong to the Hydrolase_4 superfamily except for pMGL which sequence is still unknown. These bacterial MGLs showed different substrate specificities toward MGs, di- and tri-acylglycerols, and *p*-nitrophenyl (*p*NP)-acylesters. bMGL and LipS had the highest hydrolytic activity toward monolauroylglycerol (C12:0), pMGL and MSMEG_0220 preferred monomyristoylglycerol (C14:0), Rv0183 preferred both monodecanoylglycerol (C10:0) and monolauroylglycerol (C12:0), and GMGL preferred monostearoylglycerol (C18:0). Among the reported bacterial MGLs, only Rv0183 could hydrolyze both di- and tri-acylglycerols (Cotes et al., 2007). For the synthetic *p*NP-acylester substrates, bMGL and GMGL showed the highest activity toward *p*NPC4 (Kitaura et al., 2001; Tang et al., 2019), whereas LipS preferred *p*NPC8 (Riegler-Berket et al., 2018). All reported bacterial MGLs are thermostable enzymes. Among them, while Rv0183 and MSMEG_0220 are mesophilic enzymes functioning at human body temperature (37°C), the others are all thermophilic enzymes with an optimum temperature for activity at 60–75°C, consistent with their origin from warm terrestrial/marine environments.

Monoacylglycerol lipases from non-pathogenic bacteria play an important role in lipid catabolism and also function in detoxification processes by degrading short-chain MGs (especially C10:0 and C12:0) that are highly toxic to these organisms (Conley and Kabara, 1973; Kabara and Vrable, 1977; Rengachari et al., 2013). In addition, MSMEG_0220, secreted by non-pathogenic *M. smegmatis*, was also found to participate in bacterial cell interaction (Dhouib et al., 2010). The extracellular Rv0183 from pathogenic *M. tuberculosis* H37Rv, an ortholog of MSMEG_0220, was thought to be involved in the catabolism of host membrane lipids to enhance pathogenicity (Cotes et al., 2007). Until now, nearly all reported bacterial MGLs but GMGL are from terrestrial environments. Reports on marine MGLs are still limited.

Marine environments cover more than two-thirds of the earth surface, which are mostly under low temperature, high salts, and oligotrophy. Marine microorganisms have been reported to produce a variety of enzymes with habitat-specific characteristics to adapt to the unique marine environments. However, reported bacterial MGLs are all from warm terrestrial environments or deep-sea hydrothermal vent, which are either thermophilic or mesophilic enzymes, and none of them originates from the vast cold marine environments. Whether these marine MGLs display habitat-specific characteristics is also unknown.

*Glaciecola nitratireducens* FR1064^T^, a cold-adapted and halophilic bacterium, was isolated from a surface seawater sample collected off Jeju Island, South Korea (Baik et al., 2006; Qin et al., 2014). The genome sequence of this strain was reported by our research group in Bian et al. (2011). In this study, we identified a gene encoding a novel MGL (GnMgl, accession number WP_014107521) from the genome sequence of strain FR1064^T^, which was previously annotated as an α/β fold hydrolase. GnMgl shares quite low sequence similarities with characterized MGLs (lower than 31%). GnMgl was over-expressed in *Escherichia coli* and purified. Recombinant GnMgl could hydrolyze saturated or unsaturated long-chain MGs. Similar to its source bacterium, GnMgl is also cold-adapted and halotolerant, which may help the adaption of the strain FR1064^T^ to the cold and saline marine environment. Additionally, homologs to GnMgl are found to be abundant in various marine bacterial species. Our study on GnMgl will offer a better understanding on marine bacterial MGLs.

## Materials and Methods

### Gene Cloning and Mutagenesis

Gene *GnMgl* (accession number WP_014107521) was amplified from the genomic DNA of *G. nitratireducens* FR1064^T^ using gene-specific primers (Table 1). The amplified fragment was seamlessly ligated with the vector pET22b to construct the recombinant plasmid pET22b-*GnMgl.* Using plasmid pET22b-*GnMgl* as the template, mutants S156A, D290A, and H318A were generated by a modified QuikChange site-directed mutagenesis method (Xia et al., 2015) with partially overlapping primers containing mutations (Table 1). All the recombinant plasmids were verified by sequencing.

**TABLE 1:** Primers used in this study.

Gene product	Primer	Sequence (5′–3′)^a^
GnMgl	GnMgl-F GnMgl-R	AAGAAGGAGATATACATATGCAGTTAACGAAAGAGACAGAATT (*Nde*I) TGGTGGTGGTGGTGCTCGAGCGGTGAAAAATAACTCAGTATTTTCG (*Xho*I)
S156A	S156A-F S156A-F	AATTGCGCTTCCCATTGCGTGACAAAGCAGATGCA TGCATCTGCTTTGTCACGCAATGGGAAGCGCAATT
D290A	D290A-F D290A-R	CGCCACTACCGTGGCGGCGCCAGAATA TATTCTGGCGCCGCCACGGTAGTGGCG
H318A	H318A-F H318A-R	TGTCTTTTTCGAATAGTAACTCAGCCAATGCTTCGGGTATCAATGTTG CAACATTGATACCCGAAGCATTGGCTGAGTTACTATTCGAAAAAGACA

*^*a*^The cloning sites used are underlined and the restriction enzymes are indicated in parentheses.*

### Protein Expression and Purification

Wild-type GnMgl protein and its mutants were expressed in *E. coli* BL21 (DE3) cells and induced by the addition of 1 mM isopropyl-β-D-thiogalactopyranoside (IPTG) at 18°C for 16 h. Cells were disrupted by a JN-02C French press (JNBIO, China) in 50 mM Tris-HCl buffer (pH 8.0) containing 100 mM NaCl and 5 mM imidazole and were centrifuged at 12,000 rpm for 1 h at 4°C. The supernatant was first purified by Ni affinity chromatography (Qiagen, United States) and then by gel filtration chromatography on a Superdex 200 column (GE Healthcare, Sweden) with 10 mM Tris-HCl buffer (pH 8.0) containing 100 mM NaCl. The target protein was collected and the protein concentration was measured by the Pierce BCA Protein Assay Kit (Thermo Scientific, United States) using BSA as a standard.

### Esterase Activity Assay

Esterase activity was measured as described by Li et al. (2014). The standard assay system contained 0.02 ml of 10 mM *p*-nitrophenyl-acylesters (*p*NP-acylesters) dissolved in high-performance liquid chromatography (HPLC)-grade 2-propanol, 0.02 ml enzyme and 50 mM Tris-HCl buffer (pH 8.0) in a final volume of 1 ml. After incubation at 30°C for 5 min, the reaction was terminated by the addition of 0.1 ml 20% (w/v) SDS. The absorbance of the reaction mixture at 405 nm was measured using a SpectraMax Plus384 microplate spectrophotometer (Molecular Devices, United States). All experiments were corrected for substrate autohydrolysis. One unit of enzyme activity (U) was determined as the amount of enzyme required to release 1 μmol of *p*-nitrophenol per minute by hydrolyzing *p*NP-acylesters. Substrate specificity assays were performed with *p*NP-acylesters different in acyl chain length (C2-C16) (Sigma, United States).

### Lysophospholipase Activity Assay

Lysophospholipase activity was measured as described by Kovacic et al. (2013) with some modifications. Lysophospholipid substrates 1-palmitoyl-glycerophosphocholine (C16-GPC) and 1-stearoyl-glycerophosphocholine (C18-GPC) (Sigma, United States) were prepared as 10 mM stocks in HPLC-grade 2-propanol, respectively. The reaction mixture of 1 ml containing 0.2 mM substrate, 1.5 mM NaN_3_, 0.25% (v/v) Triton X-100, 20 mM Tris-HCl (pH 8.0) and 14 μg of enzyme was incubated at 30°C for 1 h. The reaction was terminated by the addition of 0.1 ml chloroform. The same reaction system without enzyme was used as the blank control. Released fatty acids were quantified spectrophotometrically using the NEFA-HR(2) Kit (Wako Life Sciences, United States) according to the manufacturer’s instructions. One unit of enzyme activity (U) was determined as the amount of enzyme required to release 1 μmol of free fatty acid per minute by hydrolyzing lysophospholipid substrates.

### MGL Activity Assay

Monoacylglycerol lipase activity was determined as described by Aschauer et al. (2016) with some modifications. 1(3)-rac MGs were used because MGLs are not regioselective. MG substrates with different chain lengths and saturations were purchased from Sigma (United States) and prepared as 20 mM stocks in HPLC-grade 2-propanol, including 1-monolauroyl-rac-glycerol (C12:0), 1-monomyristoyl-rac-glycerol (C14:0), 3-monopalmitoyl-sn-glycerol (C16:0), 1-monostearoyl-rac-glycerol (C18:0), 1-monooleoyl-rac-glycerol (C18:1) and 1-monolinoleoyl-rac-glycerol (C18:2). The reaction mixture contained 0.1 ml of 20 mM MG substrate, 0.1 ml of 50 mM 3-[(3-cholamidopropyl)dimethylammonio]-1-propanesulfonate (CHAPS), 0.02 ml enzyme and 100 mM Tris-HCl buffer (pH 8.0) in a final volume of 1 ml. The same reaction system with 0.02 ml of buffer instead of enzyme was used as the blank control. After incubation at 30°C for 1 h, the reaction was stopped by the addition of 0.1 ml chloroform. Released fatty acids were quantified spectrophotometrically using the NEFA-HR(2) Kit (Wako Life Sciences, United States). One unit of enzyme activity (U) was determined as the amount of enzyme required to release 1 μmol of free fatty acid per minute by hydrolyzing MG substrates.

### Triacylglycerol Hydrolase Activity Assay

Triacylglycerol hydrolase activity was determined by the titration method using triacetate, tributyrin, tricaprylin, and trilaurin as substrates. The triacylglycerol emulsions were prepared in 2.5 mM Tris-HCl (pH 7.0) containing 100 mM NaCl and 1% (w/v) gum arabic by mechanical stirring. Before starting the reaction, the emulsion pH was adjusted to 7.00 by adding 10 mM NaOH. The reaction mixture contained 2 ml triacylglycerol emulsion and 0.05 ml enzyme. The same reaction system with 0.05 ml of buffer instead of enzyme was used as the blank control. After incubation at 30°C for 30 min with mechanical stirring at 180 rpm, reaction was terminated by the addition of 0.5 ml ethanol. The release of fatty acids was measured by a pH titrator (Mettler Toledo, Switzerland). One unit of enzyme (U) was defined as the amount of enzyme required to release 1 μmol of fatty acid per minute by hydrolyzing triacylglycerol substrates.

### Biochemical Characterization

By using *p*-nitrophenyl hexanoate (*p*NPC6) as substrate, the biochemical characteristics of GnMgl were studied. The optimal temperature was determined in a range from 0 to 60°C. For thermostability assay, the enzyme was incubated at 30, 35, and 40°C for different periods, and the residual activity was measured at 30°C. The optimal pH was determined with Britton-Robinson buffer in a range from pH 6.0 to 12.0 at 30°C. For pH stability assay, the enzyme was incubated in a range of pH 3.0 to 12.0 at 0°C for 1 h, and the residual activity was measured at pH 8.0 and 30°C. The effect of NaCl on enzyme activity was determined at different NaCl concentrations in a range from 0 to 4.825M at 30°C. For salt-tolerance assay, the enzyme was incubated in buffers with different NaCl concentrations ranging from 0 to 4.5M at 4°C for 24 h, and the residual activity was measured at pH 8.0 and 30°C.

The effects of metal ions (K^+^, Li^+^, Na^+^, Ba^2+^, Ca^2+^, Co^2+^, Cu^2+^, Mg^2+^, Mn^2+^, Ni^2+^, and Zn^2+^) and potential inhibitors (β-mercaptoethanol, DTT, Thiourea, Urea, EDTA, PMSF, JZL184, and *N*-arachidonoylmaleimide) on enzyme activity were tested at a final concentration of 1 or 10 mM. The effects of Tween 20, Tween 80, and Triton X-100 on enzyme activity were measured at a final concentration of 0.001, 0.01, or 0.1% (v/v). The effect of SDS on enzyme activity was examined in a final concentration of 0.001, 0.01, or 0.1% (w/v). The effects of organic solvents including methanol, ethanol, 2-propanol, dimethylsulfoxide (DMSO), dimethylformamide (DMF), acetonitrile, and acetone were tested in a final concentration of 10 or 20% (v/v).

Circular dichroism (CD) spectra of wild-type GnMgl and its mutants were recorded at 25°C on a J-810 spectropolarimeter (JASCO, Japan). All the spectra were collected from 200 to 250 nm at a scanning rate of 200 nm/min with a path length of 0.1 cm. Proteins for CD spectroscopy assays were at a concentration of 0.1 mg/ml in 50 mM Tris-HCl buffer (pH 8.0).

### Sequence Analysis

Multiple-sequence alignment was carried out using MUSCLE (Edgar, 2004). Phylogenetic analysis of GnMgl and its homologous sequences was performed using the MEGA 7.0 (Kumar et al., 2016). SignalP 4.0 (Petersen et al., 2011) was used to identify potential signal peptide sequences. PSORTb 3.0 (Yu et al., 2010) was used to predict the location of GnMgl in bacterial cells. The putative secondary structures of GnMgl was predicted by psipred (Buchan et al., 2013).

### Abundance Analysis of GnMgl in Marine Bacteria

Bacterial genomes from defined marine environments were identified from NCBI GenBank, and a total of 900 marine bacterial genomes were downloaded. By BLAST analysis against marine bacterial genomes using GnMgl as the query sequence, hits with E-values ≤ 1e^–50^, ≥ 30% sequence identity and ≥ 50% coverage in length were probed.

## Results

### Sequence Analysis of the α/β Hydrolase GnMgl From Marine Bacterium *Glaciecola nitratireducens* FR1064^T^

A gene encoding an α/β hydrolase (accession number WP_014107521), designated *GnMgl*, was identified from the genome sequence of marine bacterium *G. nitratireducens* FR1064^T^. *GnMgl* is 1,026 bp in length, encoding a putative lipolytic enzyme of 341 amino acid residues with a calculated molecular mass of 38.9 kDa and a theoretical isoelectric point of 6.54. GnMgl contains a Hydrolase_4 domain based on BLAST analysis against the Conserved Domains Database (CDD) and Pfam protein domain database. GnMgl may lack an N-terminal signal peptide sequence according to the SignalP 4.0 prediction. In addition, GnMgl is predicted to be a membrane protein by PSORTb 3.0.

GnMgl shows the highest sequence identity to an α/β hydrolase from *Glaciecola* sp. 33A (WP_101182205, 79% identity) and an uncharacterized lysophospholipase from *Glaciecola pallidula* (WP_006011195, 79% identity). Among the characterized enzymes, GnMgl was most closely related to the human monoacylglycerol lipase (3HJU) (Labar et al., 2010) with a sequence identity of only 31%, covering 45% of GnMgl sequence.

### Phylogenetic Analysis of GnMgl

**FIGURE 1:**
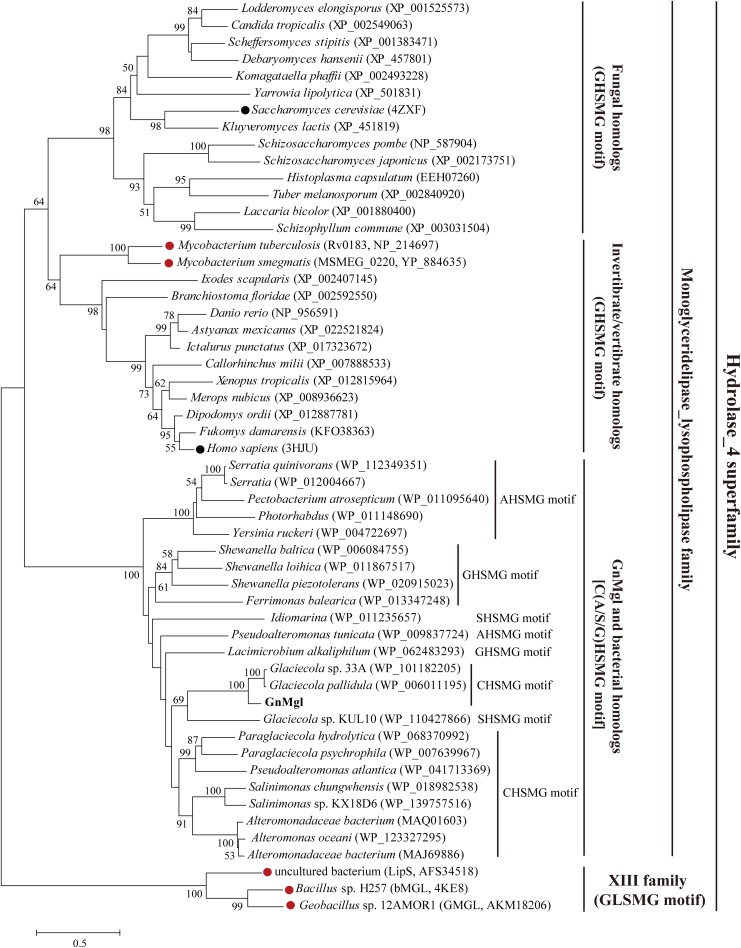
Phylogenetic analysis of GnMgl and its bacterial and eukaryotic homologs. The tree was built by the Neighbor Joining method with a JTT-matrix-based model using 209 amino acid positions. Bootstrap analysis of 1,000 replicates was conducted and values above 50% are shown. The scale for branch length is shown below the tree. Characterized monoacylglycerol lipases (MGLs) from eukaryotes and bacteria are indicated by black and red circles, respectively. The conserved pentapeptide motif containing the catalytic Ser residue is also shown for each sequence.

Homology searches against the ESTHER database suggested that GnMgl is affiliated with the monoglyceridelipase_lysophospholipase family of the Hydrolase_4 superfamily. To reveal the phylogenetic position of GnMgl in the monoglyceridelipase_lysophospholipase family, a phylogenetic tree was constructed with sequences of GnMgl and its homologs from other bacteria, fungi, invertebrates, and vertebrates (Figure 1). In this tree, GnMgl and its bacterial homologs are clustered as a separate group in the monoglyceridelipase_lysophospholipase family. As shown in Figure 1, bacterial homologs, fungal homologs, and invertebrate/vertebrate homologs are grouped into three different branches in the phylogenetic tree, suggesting that enzymes from this family possibly differentiate in these evolutionarily distant organisms. Notably, the two human-associated mycobacterial MGLs, Rv0183 and MSMEG_0220, are clustered within the invertebrate/vertebrate branch (Figure 1).

**FIGURE 2:**
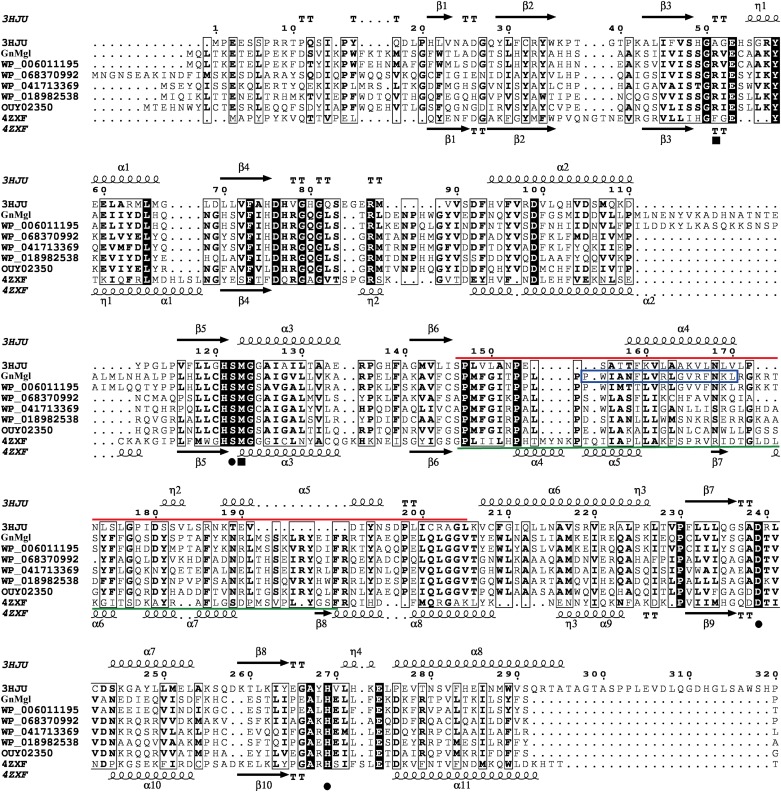
Multiple-sequence alignment of GnMgl with its bacterial and eukaryotic homologs. The two reported MGLs with structures are from human (PDB code 3HJU) and yeast (PDB code 4ZXF), respectively. Using ESPript, secondary structures of human MGL are shown above alignment and secondary structures of yeast MGL under alignment. Helices are indicated by springs, strands by arrows, turns by TT letters, and 3_10_-helices by η letters. Identical residues are shown in white on a black background, and similar residues are in bold black. Solid circles represent catalytic residues, and solid squares represent oxyanion hole residues. The cap domain of human MGL is labeled by a red line, and the cap domain of yeast MGL by a green line. The putative helix in GnMgl corresponding to the lipophilic helix α4 of human MGL is boxed in blue.

Multiple sequence alignment suggested that GnMgl has a catalytic triad possibly formed by Ser156, Asp290, and His318 (Figure 2). The potential catalytic Ser156 of GnMgl is located in the CHSMG motif, different from the typical GxSxG motif in other reported MGLs (Figures 1, 2). The oxyanion hole of GnMgl is likely composed of residues Arg58 and Met157 (Figure 2). Multiple sequence alignment combined with psipred prediction suggested that the hydrophobic helix α4 in the cap domain of human MGL is conserved in GnMgl, in spite of its low homology with mammalian MGLs (Figure 2). In human MGL, residues Cys201 and Cys208 in the cap domain and Cys242 near the catalytic site are reported to regulate the enzymatic activity (Scalvini et al., 2016). However, the counterparts of these regulatory cysteine residues are all replaced by non-cysteine residues in GnMgl (Figure 2), implying that GnMgl may be different from mammalian MGLs in catalysis.

### Expression and Purification of GnMgl

**FIGURE 3:**
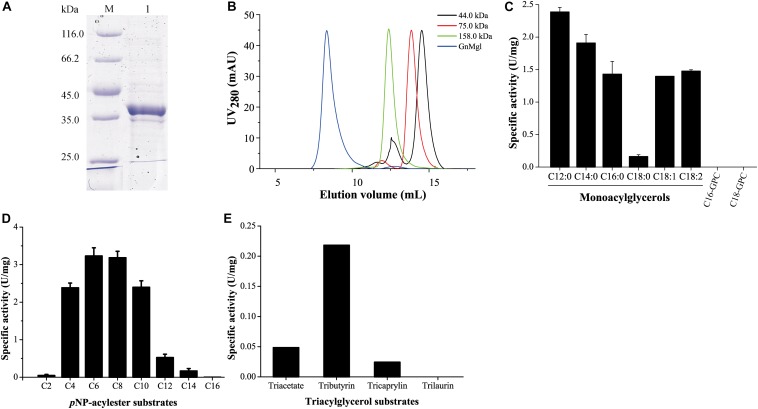
Substrate specificity of GnMgl. **(A)** SDS-PAGE analysis of purified GnMgl. Lane M, protein mass markers; lane 1, purified GnMgl. **(B)** Gel filtration analysis of GnMgl. GnMgl monomer has a calculated molecular mass of 38.9 kDa. The three protein size markers are ovalbumin (44.0 kDa), conalbumin (75.0 kDa), and aldolase (158.0 kDa). **(C)** GnMgl activity against monoacylglycerols and lysophospholipid substrates. **(D)** GnMgl activity against *p*NP-acylesters. **(E)** GnMgl activity against triacylglycerols. The graphs in **(C–E)** show data from triplicate experiments (mean ± SD).

GnMgl was over-expressed in *E. coli* BL21 (DE3), and the resulting recombinant protein was first purified by Ni affinity chromatography and then eluted by gel filtration chromatography. Sodium dodecyl sulfate polyacrylamide gel electrophoresis (SDS-PAGE) showed that the purified GnMgl exhibits an apparent molecular mass of approximately 39 kDa (Figure 3A), consistent with the calculated molecular mass of GnMgl. Gel filtration analysis suggested that, like the human MGL (residues 1–303) (Schalk-Hihi et al., 2011), the purified GnMgl tends to form aggregates in solution even in the presence of the detergents such as DDM (Figure 3B).

### GnMgl Functions as a Monoacylglycerol Lipase

Phylogenetic analysis showed that GnMgl belongs to the monoglyceridelipase_lysophospholipase family (Figure 1), suggesting that GnMgl may have MGL and/or lysophospholipase activity. Therefore, the enzymatic activity of GnMgl was assayed against monoacylglycerols, lysophospholipids, *p*NP-acylesters, and triacylglycerols (Figure 3). GnMgl had no detectable activity toward 1-palmitoyl-glycerophosphocholine (C16-GPC) and 1-stearoyl-glycerophosphocholine (C18-GPC) (Figure 3C), typical substrates for lysophospholipases, indicating that GnMgl is not a lysophospholipase. However, GnMgl could hydrolyze saturated MGs with chain length of 12–16 carbon atoms and unsaturated monostearoylglycerol (C18:0) containing one or two double bonds (Figure 3C), indicating that GnMgl acts as a MGL. Among the tested MG substrates, GnMgl showed the maximum activity toward monolauroylglycerol (C12:0) with a specific activity of 2.4 U/mg (Figure 3C). Like other MGLs (Riegler-Berket et al., 2018; Tang et al., 2019), GnMgl could hydrolyze *p*NP-acylesters (*p*NPC4-*p*NPC14), with the highest activity toward *p*NPC6 and *p*NPC8 (∼3.2 U/mg) (Figure 3D). In addition, GnMgl showed weak hydrolytic activity toward short-chain triacylglycerols (0.02–0.22 U/mg) (Figure 3E).

### GnMgl Is a Cold-Active and Alkaline Enzyme

**FIGURE 4:**
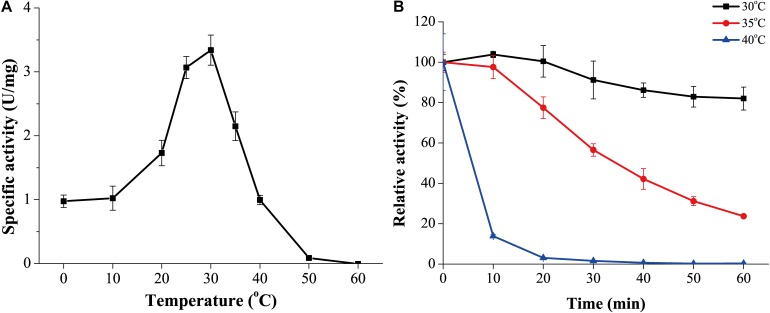
Effect of temperature on the activity and stability of GnMgl. **(A)** Effect of temperature on GnMgl activity. **(B)** Effect of temperature on the stability of GnMgl. The residual activity of GnMgl was measured after incubation at different time and temperatures. The activity of GnMgl at 0°C (3.10 ± 0.14 U/mg) was defined as 100%. The graphs show data from triplicate experiments (mean ± SD).

The optimum temperature for GnMgl activity was 30°C (Figure 4A). At 0°C, GnMgl was still active, retaining 30% of the maximum activity. GnMgl activity was sharply decreased at temperatures over 35°C and completely lost at 50°C. For thermostability, GnMgl retained more than 80% of its highest activity after 1 h incubation at 30°C (Figure 4B). GnMgl became unstable at temperatures over 30°C, losing a half of its activity after 30 min incubation at 35°C and over 80% activity after 10 min incubation at 40°C (Figure 4B). Our results above suggested that GnMgl is a cold-adapted enzyme and stable at temperatures lower than 30°C.

**FIGURE 5:**
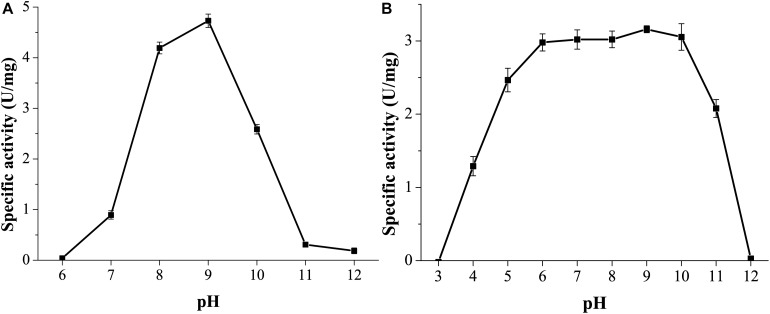
Effect of pH on the activity and stability of GnMgl. **(A)** Effect of pH on GnMgl activity. **(B)** Effect of pH on the stability of GnMgl. The residual activity was measured at pH 8.0 and 30°C after incubation at different pH for 1 h. The graphs show data from triplicate experiments (mean ± SD).

GnMgl was active at pH values between 7.0 and 11.0, and the optimum pH was 9.0 (Figure 5A), suggesting that GnMgl acts as an alkaline enzyme, consistent with its marine origin. GnMgl was stable in a wide pH range of 5.0 to 11.0 (Figure 5B). After incubation for 1 h, GnMgl retained over 66% activity in the pH range of 5.0 to 11.0.

### GnMgl Is a Halotolerant Enzyme

**FIGURE 6:**
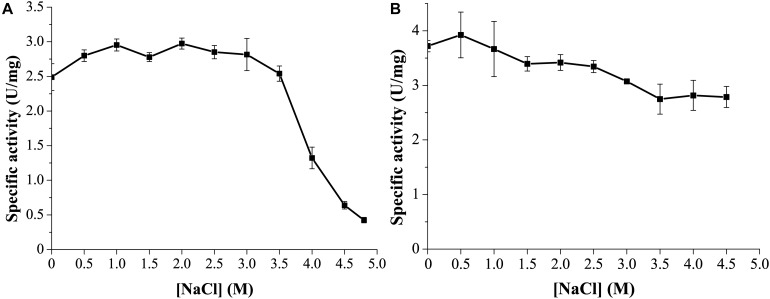
Effect of NaCl on the activity and stability of GnMgl. **(A)** Effect of NaCl on GnMgl activity. **(B)** Effect of NaCl on the stability of GnMgl. The residual activity was measured after incubation at different concentrations of NaCl and 4°C for 24 h. The graphs show data from triplicate experiments (mean ± SD).

Marine environments usually contain ∼3.5% (w/v) NaCl. Moreover, the marine strain *G. nitratireducens* FR1064^T^ where GnMgl comes from was found to be halophilic, requiring 2–9% (w/v) sea salts (optimum of 4–7%) for growth (Baik et al., 2006). Therefore, the effect of NaCl on the activity and stability of GnMgl was investigated. GnMgl still had full activity in NaCl at a concentration of as high as 3.5M (Figure 6A), indicating that GnMgl has high salt tolerance. Furthermore, after 24 h incubation in 4.5M NaCl, GnMgl still retained 75% activity (Figure 6B). These results show that GnMgl is a halotolerant enzyme.

### GnMgl Is Tolerant to Metal Ions, Detergents, and Organic Solvents

Among all the tested metal ions, only 10 mM of Cu^2+^ or Zn^2+^ severely inhibited GnMgl activity, whereas the other metal ions almost had no effect on GnMgl activity (Table 2). EDTA had no effect on GnMgl activity, indicating that the catalysis mediated by GnMgl may not require metal ions (Table 3). Like other MGLs, GnMgl was strongly inhibited by 10 mM PMSF, suggesting that GnMgl is possibly a serine hydrolase. GnMgl was also partially inhibited by 1 mM JZL184, a selective MGL inhibitor, by approximately 25% activity. Different from human MGL which is inhibited by *N*-arachidonoylmaleimide (NAM), a selective sulfhydryl-reactive MAGL inhibitor, with nanomolar level by targeting Cys201 and Cys242 (Zvonok et al., 2008; King et al., 2009; Laitinen et al., 2014), the MGL activity of GnMgl is hardly impacted by NAM with a concentration as high as 1 mM. The inhibition results combined with sequence analysis (Figure 2) suggest that the role of cysteine residues in the catalysis by GnMgl may be different from those of mammalian MGLs. GnMgl showed high resistance to reductants DTT and β-mercaptoethanol, chaotropic agents urea and thiourea, and detergents Tween 20 and Triton X-100 (Tables 3, 4). However, GnMgl was completely inactivated by 0.01% (w/v) SDS (Table 4). In addition, GnMgl showed good tolerance to all the tested organic solvents at 10% (v/v) concentration except for acetonitrile (Table 5).

**TABLE 2:** Effect of metal ions on GnMgl activity^a^.

Cations	Specific activity (U/mg)	
	1 mM	10 mM
K^+^	3.63 ± 0.08(98.3%)	2.96 ± 0.16(80.2%)
Li^+^	3.37 ± 0.33(91.4%)	3.21 ± 0.17(86.9%)
Na^+^	3.41 ± 0.17(92.3%)	3.76 ± 0.27(98.6%)
Ba^2+^	3.55 ± 0.06(96.3%)	3.49 ± 0.23(94.7%)
Ca^2+^	3.41 ± 0.14(92.5%)	3.42 ± 0.08(92.8%)
Co^2+^	3.49 ± 0.07(94.5%)	3.84 ± 0.07(104.2%)
Cu^2+^	3.21 ± 0.20(87.1%)	0.13 ± 0.01(3.4%)
Mg^2+^	3.20 ± 0.12(86.8%)	2.97 ± 0.19(80.6%)
Mn^2+^	3.28 ± 0.23(88.9%)	3.60 ± 0.08(97.5%)
Ni^2+^	3.46 ± 0.13(93.7%)	2.47 ± 0.13(67.0%)
Zn^2+^	2.52 ± 0.08(68.3%)	1.13 ± 0.02(30.6%)

*^*a*^The activity of GnMgl without the addition of metal ions (3.69 ± 0.10 U/mg) was defined as 100%.*

**TABLE 3:** Effect of potential inhibitors on GnMgl activity^a^.

Solvents	Specific activity (U/mg)	
	1 mM	10 mM
Urea	3.94 ± 0.16(106.9%)	3.74 ± 0.32(101.3%)
Thiourea	3.98 ± 0.10(107.9%)	4.02 ± 0.21(108.9%)
β-Mercaptoethanol	3.98 ± 0.10(107.9%)	4.31 ± 0.35(116.7%)
DTT	4.24 ± 0.18(114.9%)	5.00 ± 0.27(135.4%)
EDTA	3.29 ± 0.50(89.1%)	3.68 ± 0.25(99.6%)
PMSF	2.77 ± 0.28(75.1%)	1.53 ± 0.03(41.4%)

*^*a*^The activity of GnMgl without the addition of chemicals (3.68 ± 0.35 U/mg) was defined as 100%.*

**TABLE 4:** Effect of detergents on GnMgl activity^a^.

Detergent	Specific activity (U/mg)		
	0.001% (v/v)	0.01% (v/v)	0.1% (v/v)
Tween 20	3.76 ± 0.22(109.8%)	4.07 ± 0.17(118.7%)	4.37 ± 0.11(127.4%)
Tween 80	3.99 ± 0.15(116.5%)	3.36 ± 0.16(98.1%)	2.16 ± 0.02(63.1%)
Triton X-100	4.05 ± 0.16(118.0%)	3.84 ± 0.13(111.0%)	3.55 ± 0.18(102.6%)
SDS^b^	3.02 ± 0.24(87.3%)	–^c^	–^c^

*^*a*^The activity of GnMgl without the addition of detergents (3.43 ± 0.15 U/mg) was defined as 100%. ^*b*^The concentrations of SDS used were presented in w/v. ^*c*^Undetectable.*

**TABLE 5:** Effect of organic solvents on GnMgl activity^a^.

Organic solvent	Specific activity (U/mg)	
	10% (v/v)	20% (v/v)
Methanol	2.94 ± 0.07(95.8%)	1.40 ± 0.06(45.6%)
Ethanol	3.80 ± 0.24(123.8%)	0.96 ± 0.09(31.3%)
2-Propanol	3.40 ± 0.21(110.8%)	0.33 ± 0.07(10.8%)
DMSO	4.06 ± 0.26(132.2%)	4.62 ± 0.13(150.5%)
DMF	3.43 ± 0.14(111.7%)	0.38 ± 0.03(12.4%)
Acetonitrile	0.52 ± 0.04(16.9%)	0.01 ± 0.01(0.3%)
Acetone	3.01 ± 0.24(98.0%)	0.17 ± 0.07(5.5%)

*^*a*^The activity of GnMgl without the addition of organic solvents (3.07 ± 0.14 U/mg) was defined as 100%.*

### Identification of the Catalytic Triad of GnMgl Based on Mutational Analysis

**FIGURE 7:**
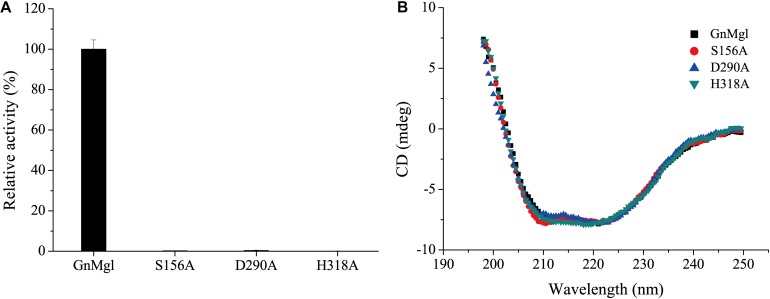
Enzymatic activities **(A)** and CD spectra **(B)** of wild-type GnMgl and its mutants. In **(A)**, reactions were conducted in triplicate in 100 mM Tris-HCl buffer (pH 8.0) containing 50 mM CHAPS at 30°C, and the concentrations of the enzyme and substrate monolauroylglycerol (C12:0) used were 0.15–0.29 μM and 2 mM, respectively. The activity of wild-type GnMgl (1.40 ± 0.01 U/mg) was defined as 100%.

Multiple sequence alignment suggested that the catalytic triad of GnMgl is most likely composed of residues Ser156, Asp290, and His318, and that the catalytic Ser156 is located in the CHSMG motif rather than in the typical GxSxG motif (Figures 1, 2). To confirm this, site-directed mutagenesis on these residues was carried out. Mutation of any of these residues to Ala led to the loss of the full activity (Figure 7A), thus confirming the key role of residues Ser156, Asp290, and His318 in catalysis by GnMgl. CD spectroscopy analysis showed that the secondary structures of the mutants exhibited little deviation from that of the wild-type GnMgl, indicating that the changes in the enzymatic activity of the mutants are caused by residue substitution rather than structural changes in GnMgl (Figure 7B).

### GnMgl Homologs Are Widespread in Marine Bacteria

**FIGURE 8:**
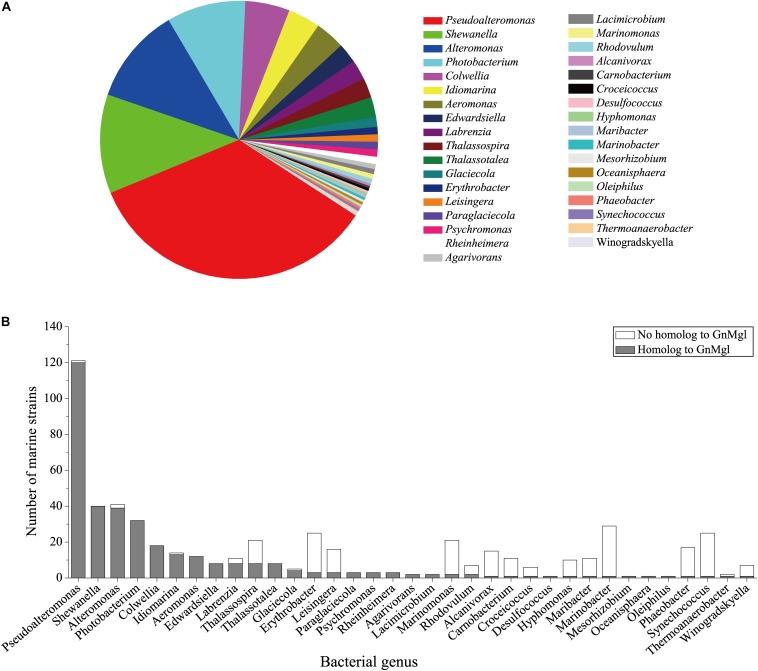
Distribution of GnMgl-like sequences in marine bacteria **(A)** and abundance of GnMgl-like sequences in each marine bacterial genus **(B)**.

To reveal the distribution of GnMgl-like sequences in marine bacteria, a total of 900 bacterial genomes with defined marine origins including seawater, marine sediment, sea ice, algae, fish, and other marine animals were downloaded from NCBI GenBank, which were affiliated with 107 genera. BLAST analysis revealed that GnMgl homologs are found in 347 marine bacterial genomes, covering 38.5% of marine bacteria. GnMgl homologs are distributed in 35 genera of *Gammaproteobacteria*, *Alphaproteobacteria*, *Deltaproteobacteria*, *Bacteroidetes*, *Firmicutes*, and *Cyanobacteria* (Figure 8), covering 32.7% of marine bacterial genera. These results show the abundance of GnMgl homologs in marine bacteria. Marine bacteria containing GnMgl-like sequences are dominated by *Alphaproteobacteria* belonging to the genera *Pseudoalteromonas* (34.6%), *Shewanella* (11.5%), *Alteromonas* (11.2%), and *Photobacterium* (9.2%) (Figure 8). Like GnMgl, most of its marine bacterial homologs contain a potential catalytic Ser residue located in unusual C(A/S)HSMG motifs (Table 6).

**TABLE 6:** Frequencies of key residues and motifs of GnMgl in the first 500 hits in NCBI nr or 347 marine bacterial homologs using BLASTP.

Residue/region types	Residue/motif	Frequency in the first 500 hits in NCBI nr^a^	Frequency in 347 marine bacterial homologs^b^
Catalytic residues	Ser156	100%	100%
	Asp290	91% (8% for Glu)^c^	98% (2% for Glu)^c^
	His318	99%	100%
Motif having catalytic Ser156	CHSMG	22%	13%
	AHSMG	43%	48%
	SHSMG	6%	7%
	A(T)HSTG	0	5%
	GHSMG	28%	27%

*^*a*^Sequence identities shared by GnMgl and its 500 homologs in NCBI nr range from 32 to 79%. ^*b*^Sequence identities shared by GnMgl and its 347 marine bacterial homologs range from 30 to 79%. ^*c*^The frequency for similar residue is shown in parentheses.*

## Discussion

Monoacylglycerol lipases are widely distributed among organisms ranging from bacteria to mammals. However, reports on bacterial MGLs are still limited and none of them is from marine environments vastly subject to low temperature, high salts, and oligotrophy. In this study, a novel MGL, GnMgl, from the marine bacterial strain *G. nitratireducens* FR1064^T^ was characterized. GnMgl is distantly related to characterized MGLs in sequence with similarity lower than 31%. Phylogenetic analysis also suggested that GnMgl and its bacterial homologs are clustered as a separate group in the monoglyceridelipase_lysophospholipase family of the Hydrolase_4 superfamily (Figure 1). In addition, different from reported MGLs with a conserved GH(L)SMG catalytic motif, GnMgl and most of its bacterial homologs harbor a catalytic Ser residue located in an atypical GxSxG motif, the conserved C(A/S)HSMG motif (Figure 1 and Table 6), suggesting that GnMgl-like enzymes might be different from reported MGLs in catalysis. Thus, GnMgl and its bacterial homologs represent a new group of MGLs.

Biochemical analysis revealed that recombinant GnMgl had no lysophospholipase activity but could hydrolyze saturated (C12:0-C16:0) and unsaturated (C18:1 and C18:2) MGs (Figure 3 and Table 7). For MG substrates, GnMgl displayed similar substrate preference to that of bMGL from *Bacillus* sp. H257 (Imamura and Kitaura, 2000; Kitaura et al., 2001). Both GnMgl and bMGL showed limited hydrolytic activity toward saturated monostearoylglycerol (C18:0), whereas other bacterial MGLs such as LipS (Riegler-Berket et al., 2018), Rv0183 (Cotes et al., 2007), MSMEG_0220 (Dhouib et al., 2010), and pMGL (Sakiyama et al., 2001) all could strongly degrade it. Furthermore, the maximum specific activity of GnMgl (2.4 U/mg) toward MGs was found to be about two orders of magnitude lower than those of other bacterial MGLs (80–620 U/mg) (Table 7). For triacylglycerol substrates, most reported bacterial MGLs showed no activity. However, GnMgl was capable of hydrolyzing short-chain triacylglycerols, which was also observed in the case of Rv0183 (Cotes et al., 2007). In conclusion, marine GnMgl is distinct from reported bacterial MGLs in both substrate selectivity and enzymatic activity.

**TABLE 7:** Comparison of biochemical characteristics between GnMgl and other bacterial MGLs.

Enzyme	Source	pH optimum	Cellular localization	Temperature optimum	Thermostability (half-life)	Activity against MG substrates (optimum)	Activity against triacylglycerols	Sequence identities to GnMgl (coverage)	References
GnMgl	*Glaciecola nitratireducens* FR1064^T^ from marine surface seawater	9.0	Cell membrane^a^	30°C	30 min at 35°C	Hydrolysis (C12:0, 2.4 U/mg)	Hydrolysis (short-chain triacylglycerols)	100% (100%)	This study
Rv0183	Human pathogen *Mycobacterium tuberculosis* H37Rv	7.5–9.0	Extracellular	50°C	<60 min at 55°C	Hydrolysis (C10:0 and C12:0, ∼330 U/mg)	Hydrolysis (long-chain triacylglycerols)	20% (93%)	Cotes et al., 2007
MSMEG_ 0220	Human non-pathogen *Mycobacterium smegmatis*	7.5–8.0	Extracellular	37°C^c^	–^b^	Hydrolysis (C14:0, 80 U/mg)	No activity	20% (81%)	Dhouib et al., 2010
bMGL	Moderately thermophilic soil bacterium *Bacillus* sp. H257	6.0–8.0	Intracellular	75°C	>10 min at 75°C	Hydrolysis (C12:0, 121 U/mg)	No activity	43% (10%)	Imamura and Kitaura, 2000; Kitaura et al., 2001
LipS	A soil metagenomic library	8.0	–^b^	70°C	>240 min at 90°C	Hydrolysis (C12:0, 105 U/mg)	No activity	45% (4%)	Chow et al., 2012; Riegler-Berket et al., 2018
pMGL	Soil bacterium *Pseudomonas* sp. LP7315	8.0–9.0	Intracellular	70°C	∼60 min at 75°C	Hydrolysis and synthesis (C14:0, 109 U/mg)^d^	No activity	–^b^	Sakiyama et al., 2001
GMGL	*Geobacillus* sp. 12AMOR1 from a deep-sea hydrothermal vent	8.0	Intracellular^a^	60°C	60 min at 70°C	Hydrolysis and synthesis (C18:0, 620 U/mg)^d^	No activity	28% (21%)	Tang et al., 2019

*^*a*^The potential cellular localization of GnMgl and GMGL was predicted by PSORTb. ^*b*^Not determined. ^*c*^The activity of MSMEG_0220 was measured at 37°C (Dhouib et al., 2010), and whether 37°C is the optimum temperature of MSMEG_0220 is unknown. ^*d*^The optimum MG substrate hydrolyzed by pMGL or GMGL was shown in parentheses.*

The marine strain FR1064^T^ where GnMgl comes from was reported to be cold-adapted and halophilic, growing at temperatures between 4 and 30°C (optimum of 25°C) within 2–9% (w/v) sea salts (optimum of 4–7%) (Baik et al., 2006; Qin et al., 2014). Biochemical characterization revealed that GnMgl displays similar characteristics to its source strain. GnMgl had the maximum activity at 30°C and retained 30% activity at 0°C. GnMgl is a cold-adapted enzyme, different from reported bacterial MGLs which are all thermophilic/mesophilic enzymes (Table 7). GnMgl is also a halotolerant enzyme, whose activity was not affected by 3.5M NaCl. The cold-adapted and salt-tolerant characteristics of GnMgl may help the adaption of the strain FR1064^T^ to the cold and saline marine environment.

PSORTb prediction suggests that GnMgl is most likely a membrane protein. The hydrophobic helix α4 in the cap domain was proposed to allow human MGL to anchor in the membrane in order to recruit its lipid substrates (Labar et al., 2010). Interestingly, the counterpart of this lipophilic helix is also found in GnMgl based on multiple sequence alignment and psipred prediction (Figure 2), which might play similar role in GnMgl as in human MGL. Moreover, the substrate preference of GnMgl correlates to the most abundant fatty acids (Baik et al., 2006) within the strain FR1064^T^. Thus, GnMgl is thought to play roles in the lipid catabolism and membrane lipid remodeling in this marine bacterium. GnMgl showed the maximum activity toward monolauroylglycerol (C12:0) which is highly toxic to bacteria, suggesting that GnMgl may also function in bacterial detoxification process. Furthermore, homologs to GnMgl are found to be abundant in various marine bacteria, suggesting that they may play an important physiological role in these marine bacteria. Therefore, our study will shed light on marine MGLs and their physiological roles in marine bacteria, and additional studies are required to further illustrate the function of GnMgl-like proteins in lipid catabolism and/or detoxification of their source strains through gene overexpression or deletion/reduction.

## Data Availability Statement

The protein sequence of GnMgl can be found in the GenBank WP_014107521.

## Author Contributions

Y-ZZ and X-YS designed the research. X-LC and P-YL directed the research. P-YL, Y-QZ, YZ, and W-XJ performed the experiments. L-SZ, Z-ZS, Y-JW, Y-SZ, and C-YL helped in data analysis. P-YL and Y-QZ wrote the manuscript. X-LC and MS edited the manuscript.

## Conflict of Interest

The authors declare that the research was conducted in the absence of any commercial or financial relationships that could be construed as a potential conflict of interest.
